# Clinical Implications of Sleep Disordered Breathing in Acute Myocardial Infarction

**DOI:** 10.1371/journal.pone.0088878

**Published:** 2014-02-11

**Authors:** Doron Aronson, Morad Nakhleh, Tawfiq Zeidan-Shwiri, Michael Mutlak, Peretz Lavie, Lena Lavie

**Affiliations:** 1 Department of Cardiology, Rambam Medical Center, Haifa, Israel; 2 Lloyd Rigler Sleep Apnea Research Laboratory, The Ruth and Bruce Rappaport Faculty of Medicine, Technion, Israel Institute of Technology, Haifa, Israel; San Diego State University, United States of America

## Abstract

**Background:**

Sleep disordered breathing (SDB), characterized by nightly intermittent hypoxia, is associated with multiple pathophysiologic alterations that may adversely affect patients with acute myocardial infarction (AMI). This prospective study investigated whether the metabolic perturbations associated with SDB are present when these patients develop AMI and if they affect clinical outcomes.

**Methods:**

We prospectively enrolled 180 AMI patients. SDB was defined as oxygen desaturation index (ODI) >5 events/hour based on a Watch Pat-100 sleep study. Blood samples were obtained for high-sensitivity C-reactive protein (hs-CRP) and markers of oxidative stress (lipid peroxides [PD] and serum paraoxonase-1 [PON-1] (arylesterase activity). Echocardiography was performed to evaluate cardiac dimensions and pulmonary artery systolic pressure.

**Results:**

SDB was present in 116 (64%) patients. Hs-CRP levels, PD and PON-1 were similar in patients with and without SDB. Echocardiography revealed higher left atrial dimension (4.1±0.5 vs 3.8±0.5 cm; *P* = 0.003) and a significant positive correlation between ODI and pulmonary artery systolic pressure (r = 0.41, *P*<0.0001). After a median follow up of 68 months, no significant differences were observed between the study groups with regard to clinical outcomes, including death, heart failure, myocardial infarction and unstable angina.

**Conclusion:**

There is a high prevalence of previously undiagnosed SDB among patients with AMI. SDB in the setting of AMI is associated with higher pulmonary artery systolic pressure. SDB was not associated with adverse clinical outcomes.

## Introduction

Sleep disordered breathing (SDB), characterized by nightly intermittent hypoxia and sleeps fragmentation, is a highly prevalent disease in the adult population [1]. There is increasing evidence that SDB is a risk factor for the development of coronary disease [2] and may trigger cardiovascular events [3].

SDB is associated with multiple alterations that may have an adverse impact in the setting of acute myocardial infarction (AMI). These include exacerbation of myocardial ischemia [4], sympathetic activation [5], systemic inflammation [6], endothelial dysfunction [7], oxidative stress [8] and increased platelet activation [9]. Thus, concomitant SDB may aggravate the clinical outcome of patients with AMI.

However, SDB is frequently not considered and therefore underdiagnosed in patients with AMI. Few data are available on the prevalence and clinical consequences of SDB in patients with AMI. Furthermore, there is no data regarding the impact of SDB on various metabolic abnormalities in patients with AMI. We hypothesized that the metabolic perturbations that are known to occur in patients with SDB may be also present in patients with AMI. We further sought to assess the impact of metabolic change as well as structural and functional alterations in left ventricular function on the clinical outcome of patients with AMI.

## Methods

### Patients

Patients presenting to the intensive coronary care unit with AMI and surviving the first 5 days of the event were prospectively enrolled into the study. We excluded patients with previous heart failure and patients presenting with cardiogenic shock. The ethics committee of Rambam Health Care Campus reviewed and approved the study and each patient signed an informed consent.

### Endpoints definitions

The primary endpoints of the study were cardiovascular mortality, re-admission for the treatment of new heart failure, recurrent myocardial infarction and readmission for unstable angina. Myocardial infarction was diagnosed according to the Universal Definition of Myocardial Infarction [10]. The diagnosis of heart failure was confirmed using hospital records and discharge summaries. Following hospital discharge, clinical endpoint information was acquired by reviewing the national death registry and by contacting each patient individually and independently reviewing the hospital course for major clinical events if the patient had been re-hospitalized. Each endpoint was evaluated separately and as a combined endpoint.

### Diagnosis of SDB

Sleep studies were performed several days after admission and preferably during the night of the last hospital day, using the Watch-PAT 100 ambulatory device. The watch-PAT 100 consists of a battery-powered, wrist-mounted, recording device and software for viewing and analysis of the recorded data. The wrist unit contains an actigraph to differentiate wake time from sleep time and 2 finger-mounted sensors. 1) a PAT probe (Itamar Medical Ltd., Caesarea, Israel) and 2) a pulse oximeter sensor (Nonin 8000 J, Plymouth, Minn). The watch-PAT 100 has been extensively validated against polysomnographic recordings for SDB diagnosis [11].

The apnea-hypopnea index (AHI) was defined as the number of episodes of apnea plus hypopnea per hour of sleep; ODI was defined as the number of decreases in oxyhemoglobin saturation by at least 3% divided by hours of sleep. SDB was defined as present when the ODI was >5 events/h [12].

### Blood sampling and laboratory procedures

Venous blood samples for high sensitivity CRP (hs-CRP) and markers of oxidative stress were obtained in the morning following the sleep study.

### Blood Chemistry

The lipid profile (total cholesterol, LDL and HDL cholesterol, and triglycerides), glucose, and creatinine in serum were determined by routine laboratory techniques.

### Lipid Peroxides

The lipid peroxides (PD) assay was based on the method by El-Saadani et al [13]. One milliliter of color reagent (CHOD-iodide-Merck cat. no 14106) was added to 100 µL diluted plasma samples, vortexed, and let stand for 30 minutes in the dark. Absorbance was read at 365 nm against the color reagent as the blank. Data are expressed as nmol PD/mL plasma.

### Paraoxonase 1-arylesterase Activity

Serum paraoxonase-1 (PON-1) arylesterase activity was measured spectrophotometrically at 270 nm with phenyl acetate as the substrate. The assay mixture consisted of 1 mmol/L of phenyl acetate and 0.9 mmol/L CaCl2 in 20 mmol/L Tris HCl, pH of 8.0, at 25°C. Nonenzymatic hydrolysis of phenyl acetate was subtracted from the total rate of hydrolysis. The data are presented as Units per minute per milliliter of serum. One unit of arylestrase activity is equal to 1 µmol of phenyl acetate hydrolyzed per minute per milliliter of serum [14].

### Echocardiographic studies

Echocardiography was performed during hospital stay after a median of 2 days from admission [interquartile range 1 to 3 days]. Left ventricular and left atrial dimensions were obtained using M-mode echocardiography, guided by two-dimensional imaging. Left ventricular internal dimension and interventricular septal and posterior wall thicknesses were measured at end diastole and end systole according to the American Society of Echocardiography recommendations.

Left ventricular ejection fraction (LVEF) was assessed by a combination of the Teichholz formula and visual estimation from multiple echocardiographic windows, and classified as normal (≥55%), mildly reduced (45–54%), moderately reduced (30–44%) or severely reduced (<30%).

The pulmonary artery systolic pressure (PASP) was calculated as the sum of the peak systolic pressure gradient across the tricuspid valve (using the modified Bernoulli equation), and the right atrial (RA) pressure. RA pressure was estimated according to the size and respiratory variation of the inferior vena caval diameter in the subcostal view [15].

### Statistical analysis

Data are expressed as mean ± SD or median and interquartile range. The baseline characteristics and laboratory parameters of the study groups were compared using unpaired *t* test for continuous variables and by the χ^2^ statistic for categorical variables. When continuous data was not normally distributed or had unequal variance, groups were compared with the nonparametric Mann-Whitney U test. Spearman rank-order correlations were calculated between the various biomarkers and echocardiographic data and the sleep studies data. Multivariable linear regression analysis was used to determine the relationship between ODI and biomarkers or echocardiographic parameters, adjusting for other relevant variables.

Event–free survival was estimated by the Kaplan–Meier method for each endpoint. Stepwise Cox proportional hazards models with backward selection were used to calculate hazard ratios (HRs) and 95% CI for SDB. We sought a final parsimonious model that included only those baseline variables that differed between patients with and without SDB.

Patients receiving CPAP therapy were censored at the time of therapy initiation. Differences were considered statistically significant at the 2-sided *P*<0.05 level. Statistical analyses were performed using the STATA Version 12.0 (College Station, TX).

## Results

Between February 2005 and June 2009, 220 patients with AMI were enrolled. After exclusions for technical failures (n = 40), complete sleep analyses were obtained in 150 men and 30 women. The median duration from hospital admission to sleep study was 5 days. The median ODI and AHI of the study population were 8 (interquartile range 3 to 18 events/h) and 17 events/h (interquartile range 5 to 30 events/h), respectively. One hundred sixteen patients (64%) were diagnosed as having SDB with an ODI>5/hour. All patients in which SDB was detected were notified of the sleep study results and referred to sleep medicine specialists for consultations.

The clinical characteristics of patients with and without SDB are summarized in Table 1. Patients with SDB were more likely to be older and females, and had higher prevalence of hypertension and higher BMI.

**Table 1:** Baseline Clinical Characteristics according to ODI.

	ODI≤5	ODI>5	
Characteristics	(n = 64)	(n = 116)	*P* value
Age (years)	56±11	59±9	0.03
Female gender	5 (8%)	25 (22%)	0.02
Previous infarction	10 (16%)	25 (22%)	0.34
Body mass index (Kg/m^2^)	26±3	30±6	<0.0001
Lipid profile			
Total cholesterol (mg/dl)	181±49	180±43	0.90
LDL cholesterol (mg/dl)	109±40	111±38	0.48
Triglycerides (mg/dl)	171±106	157±96	0.13
HDL cholesterol (mg/dl)	38±9	44±25	0.77
Hypertension	24 (38%)	62 (53%)	0.04
Current Smoking	22 (34%)	29 (25%)	0.18
Diabetes	13 (20%)	35 (30%)	0.15
Creatinine (mg/dl)	1.0±0.3	1.0±0.3	0.51
Killip Class II-IV	6 (10%)	16 (14%)	0.38
Anterior infarction	28 (44%)	44 (38%)	0.45
ST-elevation infarction	42 (66%)	66 (57%)	0.25
Medical therapy			
Aspirin	62 (97%)	115 (99%)	0.26
Clopidogrel	57 (89%)	106 (91%)	0.61
Beta blockers	55 (86%)	103 (89%)	0.58
ACE inhibitors/ARBs	82 (81%)	63 (80%)	0.81
Statins	61 (95%)	109 (94%)	0.71
Primary angioplasty	28 (44%)	45 (39%)	0.52
Coronary revascularization	47 (84%)	86 (85%)	0.84
Sleep parameters			
Apnea-hypopnea index (events/h)	8±5	32±21	<0.0001
ODI (events/h)	2±2	21±18	<0.0001
Minimal O^2^ Saturation (%)	89±4	83±7	<0.0001

Data are mean ± SD or number (%). Continuous variables were compared using unpaired *t* test. Categorical variables were compared by the χ^2^ statistic.

ACE  =  Angiotensin converting enzyme; ARBs  =  Angiotensin II receptor blockers.

### Effect of SDB on markers of inflammation and oxidative stress

Plasma hs-CRP levels were nonsignificantly higher in patients with SDB. Both PON1 and PD were similar among patients with and without SDB (Table 2).

**Table 2:** Markers of inflammation and oxidative stress in patients with and without SDB.

	ODI≤5	ODI>5	
Characteristics	(n = 64)	(n = 116)	*P* value
C-reactive protein (mg/L)	9.4 [5.6–29.7]	15.3 [6.2–32.6]	0.33
Lipid peroxides (nmol/mL)	925 [802–1063]	897 [795–1055]	0.49
Paraoxonase 1-arylesterase Activity (U·min·^1^ml^−1^)	76 [62–81]	71 [63–82]	0.91

### Effect of SDB on echocardiographic parameters

Left ventricular systolic and diastolic dimensions, LVEF and left ventricular wall motion score index were similar among patients with and without SDB, while left atrial dimension was larger in patients with SDB (Table 3). There was a moderate positive correlation between LA size and ODI (r = 0.39, *P*<0.001).

**Table 3:** Echocardiographic characteristics of patients with and without SDB.

	ODI≤5	ODI>5	
Characteristics	(n = 64)	(n = 116)	*P* value
Left ventricular end systolic dimension (cm)	3.6± 0.7	3.4±0.8	0.76
Left ventricular end diastolic dimension (cm)	5.1±0.5	5.2±0.6	0.22
Interventricular septal thickness (cm)	1.0±0.2	1.0±0.2	0.76
Posterior wall thickness (cm)	0.8±0.1	0.9±0.1	0.32
Left ventricular ejection fraction (%)	48±12	47±13	0.43
Left ventricular wall motion score index	1.5±0.5	1.6±0.4	0.30
Left atrial dimension (cm)	3.8±0.5	4.1±0.5	0.003
Pulmonary artery systolic pressure (mm Hg)	29±7	33±9	0.006

**Figure 1.Correlation:**
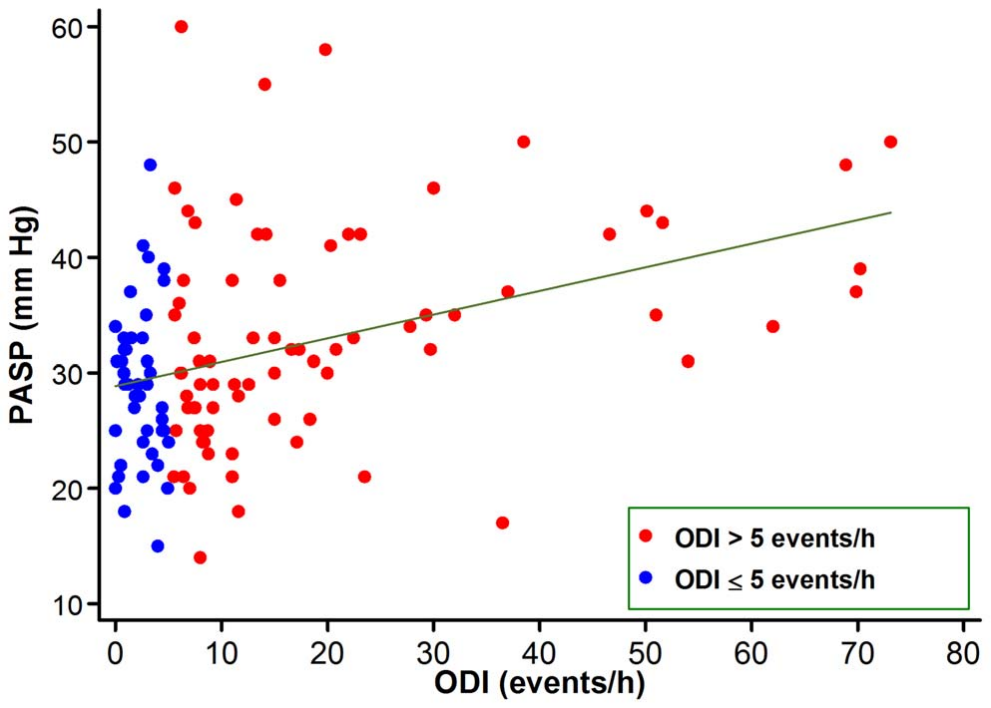
between ODI and pulmonary artery systolic pressure.

PASP was evaluable in 120 patients (71 [70%] without and 49 [62%] with SDB, respectively, *P* = 0.24). PASP was higher in patients with SDB (Table 3). There was a moderate positive correlation between ODI and PASP (r = 0.41, *P*<0.0001; Figure 1). In a multivariable linear regression model, adjusting for age, gender, BMI and left ventricular systolic function, the positive relationship between PASP and ODI remained highly significant (*P* = 0.001).

### Effect of SDB on clinical outcomes

**Figure 2:**
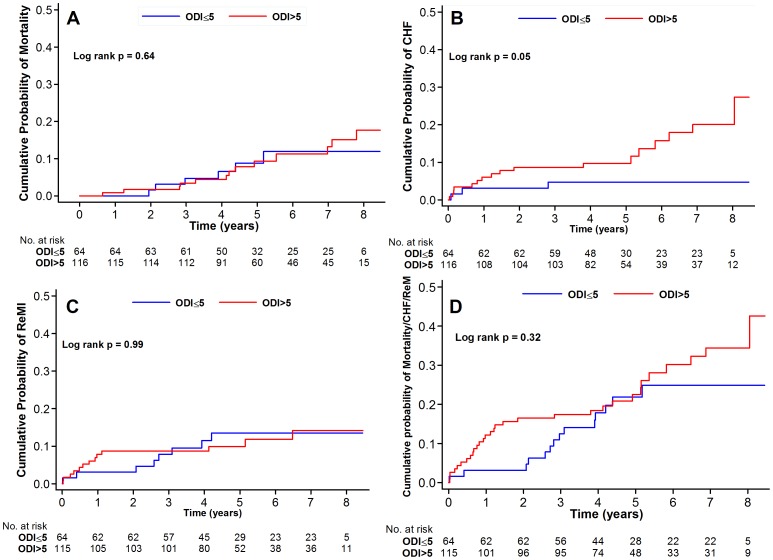
Kaplan-Meier curves for the clinical endpoints including mortality (A), readmission for heart failure (B), recurrent infarction (C) and the combined endpoint of mortality, readmission for heart failure and recurrent infarctions (D).

**Figure 3:**
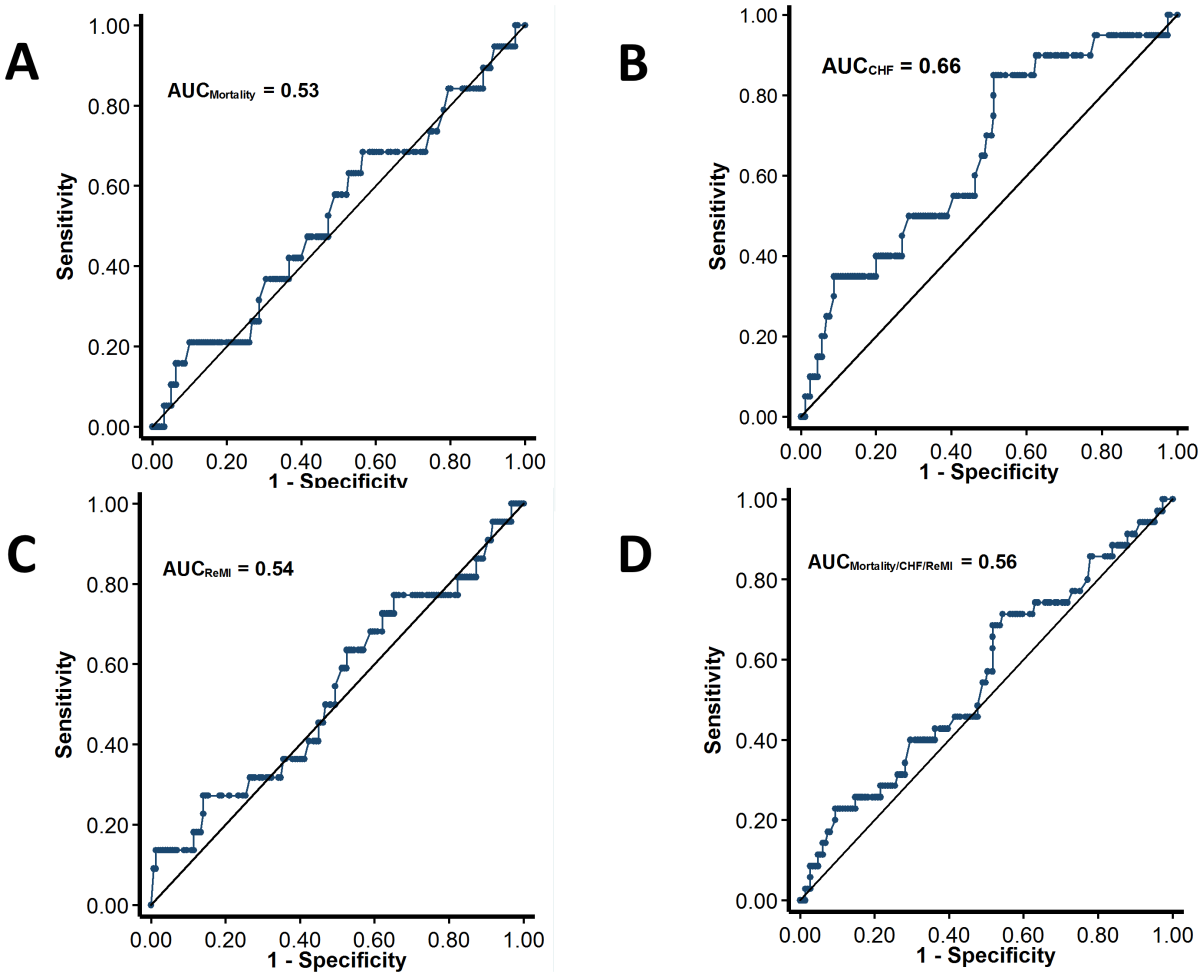
Receiver-operating characteristic curves for the performance of ODI in the prediction of mortality (A), congestive heart failure (B), recurrent infarction (C) and the combined endpoint of mortality, readmission for heart failure and recurrent infarctions (D).

The median follow-up after hospital discharge was 68 months. The rates of hard cardiovascular endpoints including death, readmission for CHF and recurrent infarctions were generally low. For the clinical outcomes analysis, 4 patients were censored at the time of CPAP therapy initiation. Figure 2 displays the Kaplan-Meier curves for the endpoint of mortality, readmission for heart failure, recurrent infarction and the combined endpoint of mortality heart failure and recurrent infarctions. Figure 3 shows the area under the ROC curves for the respective endpoints.

None of these endpoints were significantly different between patients with and without SDB with the exception of readmission for heart failure. However, after adjustments for other covariates in a Cox proportional hazards model, SDB was not an independent predictor of any of the endpoints (Table 4). Similar results were obtained in a sensitivity analysis using AHI >5 events/h for the diagnosis of SDB, and with the use of ROC-derived optimal ODI cutoffs.

**Table 4:** Unadjusted and Adjusted Cox's proportional Hazards Model for Clinical Endpoints in Patients with SDB.

	Unadjusted		Adjusted	
Endpoint	HR (95% CI)	*P* value	HR (95% CI)	*P* value
Mortality	1.19 (0.45–3.12)	0.73	0.88 (0.33–2.34)	0.79
Admission for heart failure	3.19 (0.93–10.88)	0.06	2.50 (0.71–8.77)	0.27
Recurrent infarction	0.93 (0.39–2.25)	0.88	0.94 (0.39–2.27)	0.89
Unstable Angina	1.40 (0.71–2.74)	0.33	1.38 (0.66–2.87)	0.39
Mortality/heart failure/recurrent infarction	1.34 (0.71–2.51)	0.37	1.05 (0.55–2.00)	0.15

Models were adjusted for age, gender, BMI and PASP.

## Discussion

In this prospective study we found a high prevalence of previously undiagnosed SDB in patients admitted to the hospital for AMI. There were no structural and functional cardiac alterations among patients with SDB with the exception of increased left atrial dimension. However, pulmonary artery systolic pressures were elevated among patients with SDB and correlated with its severity. Contrary to our hypothesis SDB was not associated with any discernible impact on clinical outcomes with the possible exception of heart failure.

### Prevalence of SDB in AMI patients

In the present study, 64% of patients admitted for AMI were diagnosed with SDB. Few data are available with regard to the prevalence of SDB among patients with acute coronary syndromes. However, although the method of diagnosis and definitions of SDB vary between studies, all have consistently demonstrated high prevalence of SDB ranging from 22% to 69% [16]–[21]. Similarly, in patients with stable coronary artery disease, the prevalence of SDB ranges from 30 to 54% [22], [23].

The pathophysiological effects of SDB on the cardiovascular system involve complex mechanical, hemodynamic, neurohumoral, and inflammatory mechanisms [1], [6]. The high prevalence of concomitant SDB in patients with acute coronary syndromes underscores the importance of better understanding of the consequences and clinical implications of SDB in these patients.

### Inflammation and oxidative stress

Previous studies have reported that patients with SDB have evidence of systemic inflammation—including elevated levels of CRP and pro-inflammatory cytokines such as tumor necrosis-α and interleukin-6 [24]. However, it remains controversial whether these findings depend more on obesity or sleep apnea. Oxidative stress has been hypothesized to be one of the plausible pathogenic mechanisms underlying the associations between SDB and coronary heart disease. There has been growing evidence that intermittent hypoxia (IH) and reoxygenation during repetitive SDB may elicit increased vascular oxidative stress [8], [25]. However, few data are available regarding markers of inflammation or oxidative stress in patients with SDB in the setting of AMI [12].

In contrast to previous studies in stable subjects with SDB, hs-CRP, PD and the activity of the endogenous antioxidant enzyme PON1 were not affected by the presence of SDB. A possible explanation for these findings is that serum antioxidants are not elevated in patients with myocardial infarction but rather with cardiovascular risk factors, such as cigarette smoking [26] and diabetes [27]. In addition, HMG-CoA reductase inhibitors, which can reduce reactive oxygen species [28] were used in nearly all patients in the present study, potentially masking the differences between the groups.

### Echocardiographic findings in SDB

In the general population, airway occlusion during sleep is associated with a reduced LVEF and increased LV end-systolic volume [29]. Recurrent acute increases in left ventricular volume might lead to eccentric remodeling and contribute to adverse left ventricular remodeling when superimposed on the acute ischemic injury in AMI patients. It is possible that the profound changes in loading conditions during apnea after an acute myocardial injury may impair the recovery of LV systolic function in patients after AMI [20]. However, in the present study, SDB was not associated with larger left ventricular internal dimensions or with reduced left ventricular systolic function. It is possible that the acute infarction was the dominant factor in determining left ventricular dimensions and function. By contrast, left atrial dimension was larger among patients with SDB. This finding may represent greater impairment of diastolic function in SDB patients [30], leading to left atrial enlargement.

Mild pulmonary hypertension is found in a sizable minority of patients with SDB, even in the absence of clinically recognizable lung disease or left-sided heart disease [31]. In the present study, pulmonary hypertension was higher in patients with SDB, and correlated positively with ODI. Pulmonary hypertension and SDB share common risk factors—namely, obesity and aging, which may confound risk factor associations [32]. Notwithstanding, the strong positive association between ODI and PASP remained significant after adjusting for age, sex, BMI and left ventricular systolic function. Importantly, elevated PASP after AMI frequently represents postcapillary pulmonary hypertension due to elevated capillary wedge pressures. As such, increased PASP is a marker of latent subclinical CHF and predicts the development of overt clinical CHF [15].

### SDB and clinical outcome

In the general population, SDB is associated with increased risk for major cardiovascular events including AMI, heart failure, stroke and life-threatening arrhythmias [1], [33], [34]. Therefore, it may be postulated that untreated concomitant SDB might be a risk factor for adverse outcome after AMI. Potential mechanisms underlying such an association include recurrent episodes of hypoxemia and arousal from sleep after obstructive respiratory events, both of which cause an increase in sympathetic activity and blood pressure [5] that result in increased left ventricular afterload and the propensity for arrhythmias [33]. Furthermore, the forceful inspiratory efforts generated in the face of an obstructed airway result in large negative swings in intrathoracic pressure, which consequently increases transmyocardial pressure [35]. Despite of these considerations, that provided part of the rational for the present study, we observed no difference between patients with and without SDB with regard to hard clinical endpoints, with the potential exception of incident heart failure. These results are in agreement with previous studies on patients with SDB in the setting of acute coronary syndromes [16]–[18].

The sample size of the present study might have been insufficient to demonstrate a significant association between SDB and hard cardiovascular endpoints. In addition, survival bias toward a null result may be present as only patients who survived the first several days after the infarction were recruited. Notwithstanding, given the results of the present and previous studies [16]–[18], the clinical significance of SDB in the post-infarction setting remains to be established.

Although several mechanisms may promote CHF in SDB, there is also evidence that SDB might be associated with activation of cardiovascular adaptive mechanisms [12], [36], [37]. In this context, our group has recently demonstrated the existence of IH-associated protective mechanisms that are activated in patients with AMI. Specifically, endothelial progenitor cell numbers and proliferative and angiogenic properties are heightened in patients with AMI and coexistent SDB compared with patients with AMI without SDB [12]. Similarly, the proliferative and angiogenic properties of EPCs from healthy individuals were increased after exposure to IH *in vitro*, indicating that IH associated with SDB promotes EPC numbers and functions [12].

## Conclusions

We found a high prevalence of previously undiagnosed SDB among patients admitted to the hospital with AMI. SDB in the setting of AMI is associated with higher pulmonary artery systolic pressure. SDB was not associated with adverse clinical outcomes.
